# Supplementary material for: Development and Optimal Immune Strategy of an Alum-Stabilized Pickering emulsion for Cancer Vaccines
Source: Vaccines (Basel). 2023 Jun 28;11(7):1169. doi: 10.3390/vaccines11071169 (PMC10383433; doi:10.3390/vaccines11071169)
Supplement: Supplementary file 1 [file vaccines-11-01169-s001.zip › vaccines-2437112-supplementary.pdf]

Supplementary Material

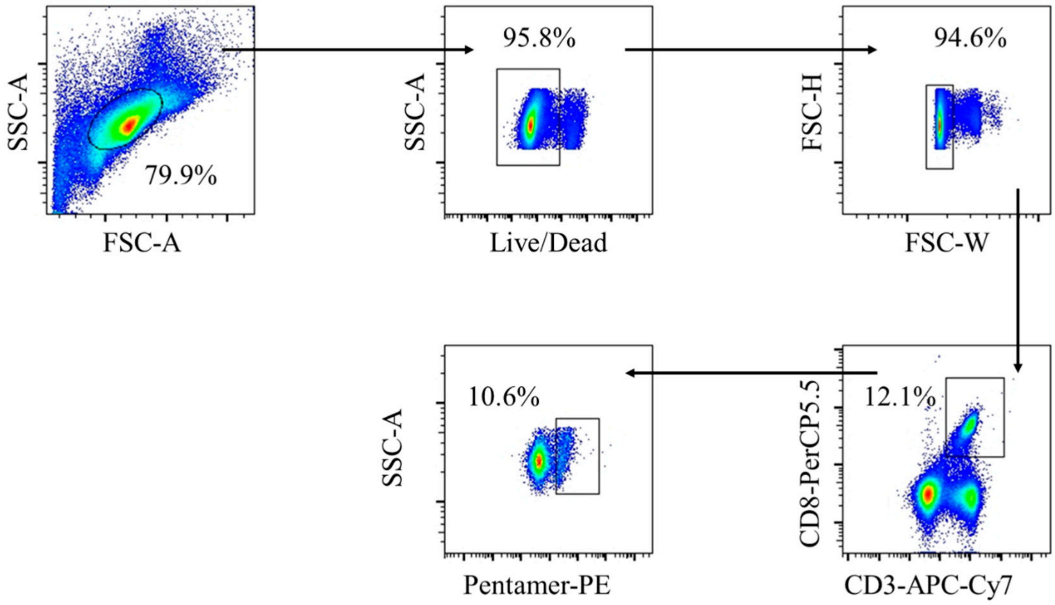

Figure S1. Gating strategy of OVA-specific $CD8^+$ T cells in the spleen.

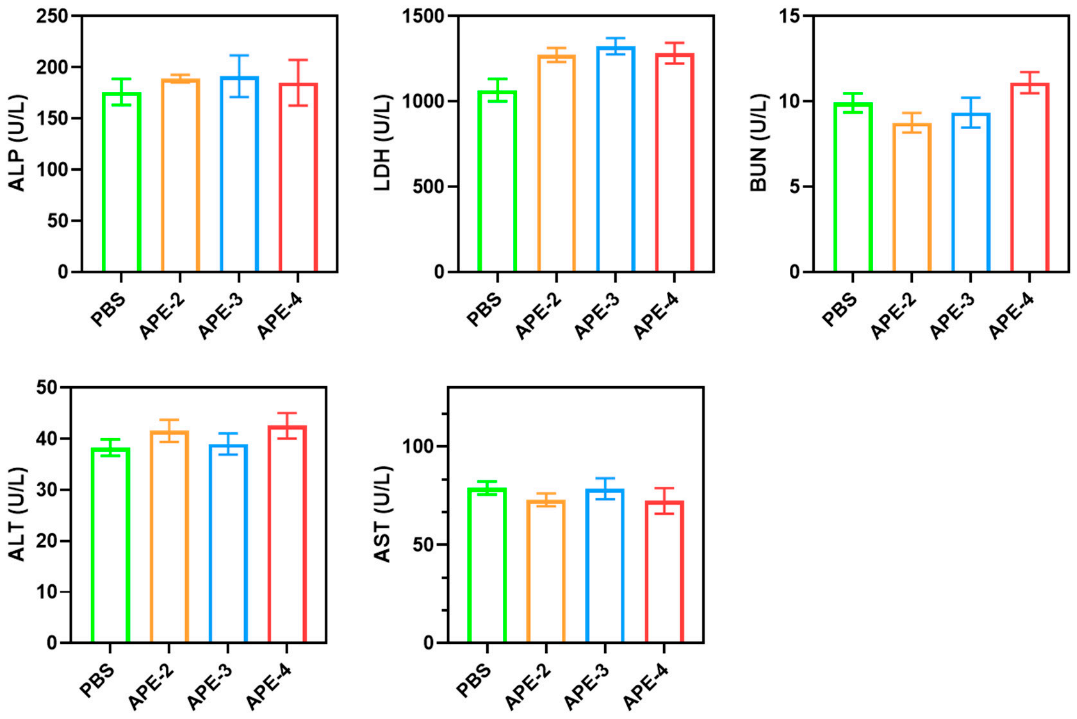

Figure S2. Serum chemistry levels in mice. Including lactate dehydrogenase (LDH), alanine aminotransferase (ALT), aspartate aminotransferase (AST) and blood urea nitrogen (BUN). Data are expressed as mean ± SEM (n = 6).

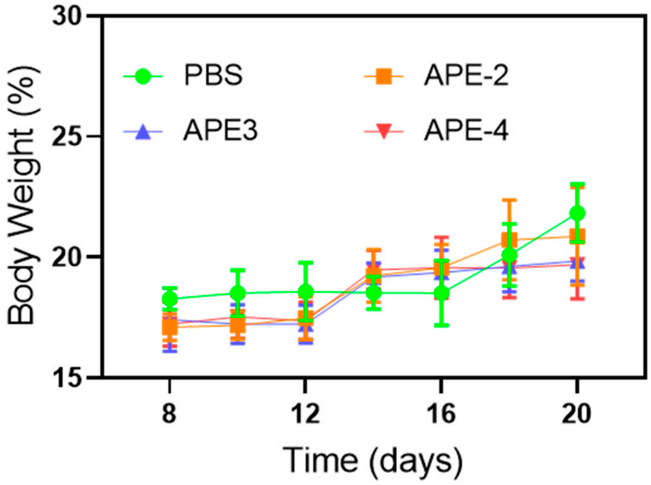

Figure S3. Body weight changes after different treatments (APE

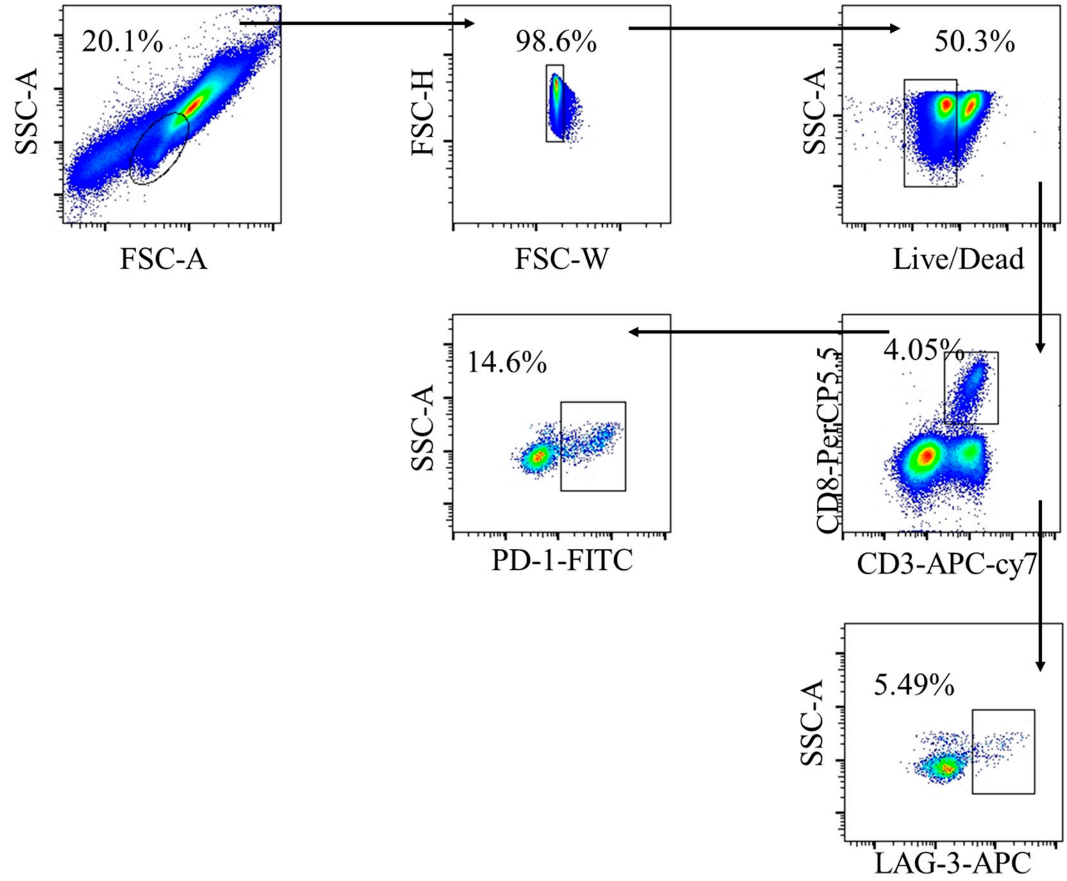

Figure S4. Gating strategy of PD-1^{+} cells or LAG-3^{+} cells in CD8^{+} T cells (CD3^{+} CD8^{+}) in TME.

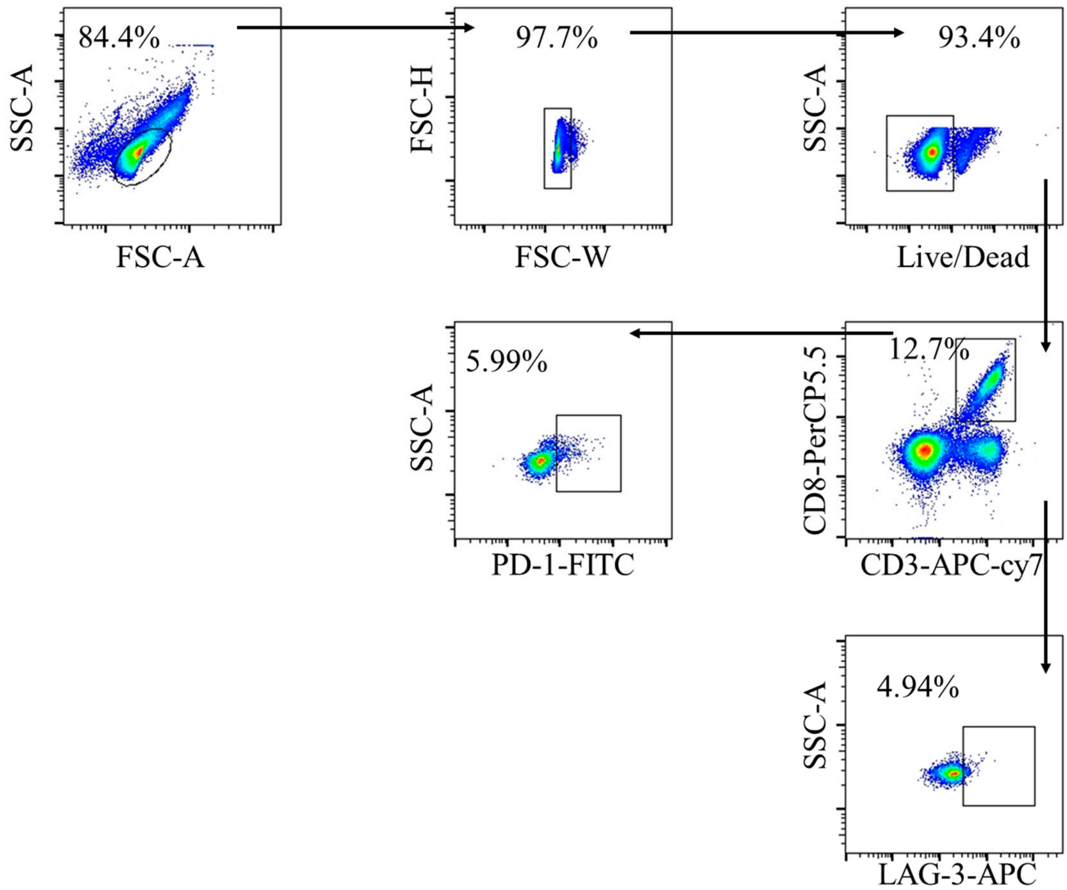

Figure S5. Gating strategy of PD-1^{+} cells or LAG-3^{+} cells in CD8^{+} T cells (CD3^{+} CD8+) in TDLNs.

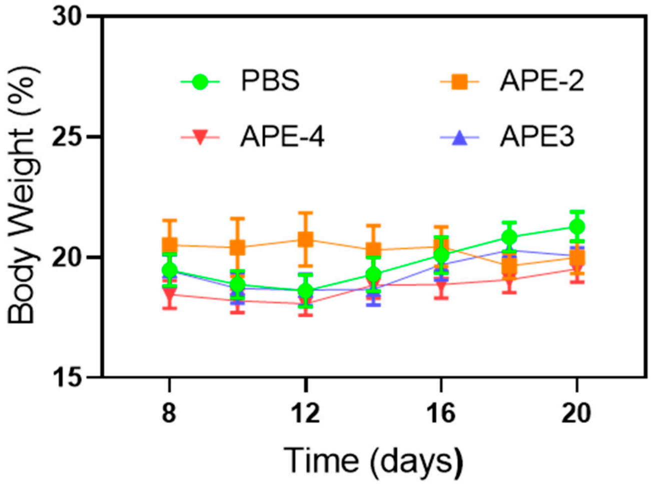

Figure S6. Body weight changes after different treatments (APE + anti-PD-1).
